# Post tubercular bronchiectasis with aspergilloma

**DOI:** 10.11604/pamj.2024.48.68.43859

**Published:** 2024-06-26

**Authors:** Ashwin Karnan

**Affiliations:** 1 Department of Respiratory Medicine, Jawaharlal Nehru Medical College, Datta Meghe Institute of Higher Education and Research, Sawangi (Meghe), Wardha, Maharashtra, India

**Keywords:** Fungus, cough, breathlessness

## Image in medicine

A 48-year-old diabetic male, presented to the outpatient department with complaints of fever, breathlessness on exertion, and cough with mucopurulent expectoration for the past 15 days. The patient gave a history of pulmonary tuberculosis 4 years back for which he took antitubercular therapy for 6 months. CT thorax showed a cavity with a hypodense lesion and cystic bronchiectatic changes in the left lower lobe. Echocardiography showed a dilated right ventricle with pulmonary arterial hypertension. Sputum for acid-fast bacilli was negative, serum IgG antibody for Aspergillus was raised and Bronchoalveolar lavage galactomannan was positive confirming the diagnosis of Aspergilloma. The patient was treated with Voriconazole, phosphodiesterase-5-inhibitor, oral corticosteroids, oral hypoglycemic agents, and diuretics. The patient improved symptomatically and is currently on follow-up. Aspergillus colonization usually occurs in a previously existing lung cavity. Aspergilloma may be difficult to diagnose due to its complexity. It has around 38% mortality rate. Hemoptysis is the dreaded complication requiring bronchial artery embolization.

**Figure 1:**
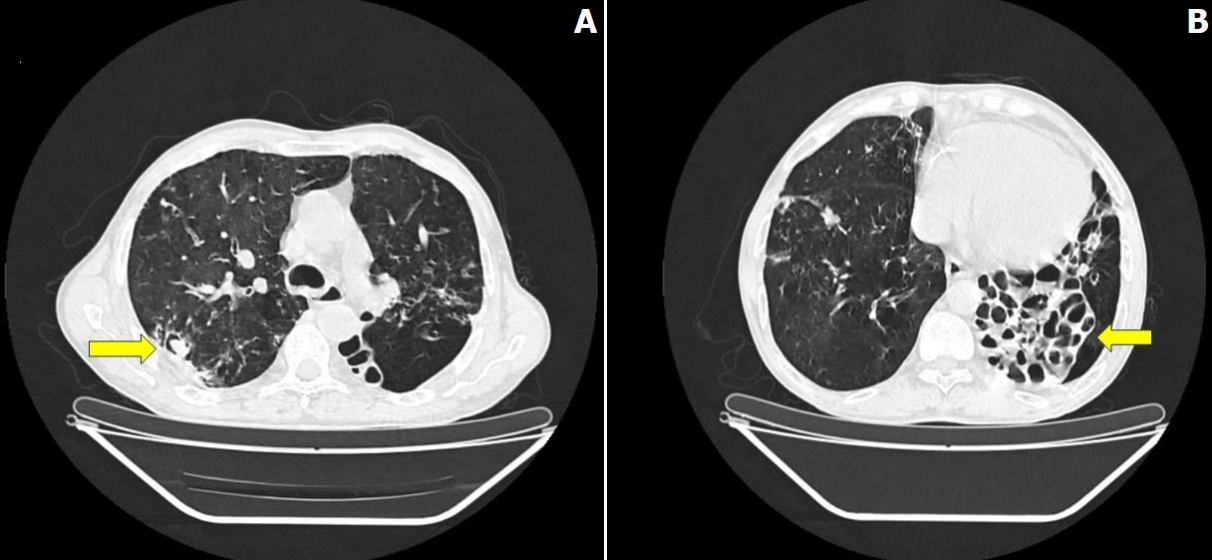
A) CT of the thorax with yellow arrow showing cavity with hypodense content depicting air crescent sign; B) CT of the thorax with yellow arrow showing cystic bronchiectasis in the left lower lobe

